# Maps of forest vertical structure for Colombia, a megadiverse country

**DOI:** 10.1038/s41597-025-06297-7

**Published:** 2025-12-03

**Authors:** J. Camilo Fagua, Patrick Jantz, Patrick Burns, Samuel M. Jantz, John B. Kilbride, Scott J. Goetz

**Affiliations:** 1 Grupo de Biodiversidad, Biotecnología y Conservación de Ecosistemas, Departamento de Biología, Facultad de Ciencias, Universidad Nacional de Colombia—Sede Bogotá, Bogotá, DC 111321 Colombia; 2 Global Earth Observation & Dynamics of Ecosystems Lab (GEODE), School of Informatics, Computing, and Cyber Systems (SICCS), Northern Arizona University, Flagstaff, AZ 86011 USA; 3 National Institute for Modeling Biological Systems (NIMBioS), University of Tennessee, Knoxville, TN 37996 USA; 4 Renoster Systems Inc, 21750 Hardy Oak Blvd Ste 104, PMB 37519, San Antonio, TX 78258-4946 USA

**Keywords:** Forestry, Ecological modelling

## Abstract

Vegetation vertical structure refers to the 3D distribution of vegetation aboveground biomass. Vegetation vertical structure of tropical forests influences other ecological and environmental variables that are essential for the functioning of the ecosystems. Integrating over 5.9 million Globel Ecosystem Dynamics Investigation (GEDI) LiDAR (Light Detection and Ranging) footprints, multispectral, and synthetic aperture radar (SAR) imagery, we built five national maps at 25 m resolution of five forest structural metrics for Colombia, South America, for the year 2020. We mapped canopy height, the height of half the cumulative returned energy from GEDI (RH50), total canopy cover, foliage height diversity, and total plant area index. The resulting maps tended to have the highest errors in the Amazon and Andean regions. Total cover had the highest relative error. Interrelationship curves between forest structural metrics of GEDI footprints are maintained across mapped metrics, indicating that the predictive models preserve structural relationships observed in GEDI data. Due to the medium-high spatial resolution and national coverage of the forest structural maps presented in this work, these maps will be useful for evaluating and mapping other ecological variables and conservation priorities in Colombia.

## Background & Summary

Three-dimensional vegetation structure (or vegetation vertical structure) refers to the distribution of plant biomass from the ground to the top of the canopy^1–3^. Vegetation vertical structure is an Essential Biodiversity Variable, a set of biological variables designed to monitor biodiversity changes in response to the current environmental crisis that the planet is experiencing^4^. Vegetation vertical structure influences hydrological cycles^5–7^, climatic regulation^8–10^, primary productivity^11–14^, nutrient fluxes^15,16^, habitat quality^17–20^, and biodiversity^21–23^. The most consistent and low-cost method to study vegetation vertical structure over large extents consists of using LiDAR (Light Detection and Ranging) sensors to estimate metrics that describe 3D vegetation structure, due to LiDAR’s ability to penetrate canopies and measure the sub-canopy distribution of vegetation^24–29^.

The NASA Global Ecosystem Dynamics Investigation (GEDI) LiDAR was designed to study vegetation vertical structure near-globally between approximately 51.6 degrees north and south latitude. It acquired data from April 2019 to March 2023, then was paused for 13 months^30,31^, and began reacquiring data in April 2024. GEDI uses eight laser beams which measure forest structure within ~25 m footprints. Along track, these footprints are spaced by 60 m, with 600 m spacing between beams^32^. Although GEDI tends to acquire fewer high-quality footprints in the tropics due to the geometrical characteristics of its orbit and persistent cloud cover^3,30^, never have there been so many detailed measurements of forest vertical structure in tropical ecosystems, the most diverse terrestrial areas on the planet^33^ and where the highest rates of natural habitat loss occur^34^.

GEDI footprints have limitations for spatially-continuous mapping because these footprint-level products represent samples of the land area, leaving most of the land surface without observations. GEDI is capable of discontinuously sampling only ~4% of the land surface every two-years^30,31^. Consequently, some research groups have integrated GEDI footprints with wall-to-wall multispectral data to enable the spatial prediction of GEDI information for consistent gridded maps of vegetation structure metrics and aboveground biomass. These predictions include a canopy height map at 30 m over the GEDI domain using Landsat predictors and RH95 as an indicator of height^35^, a global map of canopy height at 10 m using energy level RH98 and Sentinel-2 predictors^36^, maps of mean and standard deviation of canopy height at 1 km using the energy level RH100^37^, global maps of relative height metrics at 100 m, 200 m, 500 m, and 1000 m spatial resolutions integrating GEDI and ICESat2 (Ice, Cloud, and Land Elevation Satellite 2)^38^, and gridded mean aboveground biomass density at 1 km based on the canopy heights generated by GEDI^39^. This type of work modeled canopy height but did not map metrics related to the distribution of biomass between the ground and the canopy height. Burns *et al*.^3^ developed and published annual global maps from 2019 to 2023 of 26 GEDI structural metrics related to entire vertical vegetation profile at coarse spatial resolutions (1 km, 6 km, and 12 km), gridding the aggregated footprint values^3^. There are limited published maps of vegetation structure variables generated by GEDI predictions or interpolation that describe the entire vertical vegetation profile with a detailed resolution (<= 30 m) for large regions or countries. Those that have been published show great promise for enhancing our understanding of forest structure gradients and species habitat relationships^40^.

The objective of this research is to elucidate the construction and make available five maps of metrics of forest vertical structure (Table 1) with relatively high spatial resolution (25 m) for the year 2020 in Colombia, one of the most biodiverse countries on the planet. Colombia includes vegetation types that range from dry, moist, to rain forest at altitudes from sea level to >~5000 m. The maps were constructed by developing predictions for each metric of forest vertical structure using a set of 82 remote sensing predictors (temporal metrics) that included data from multispectral (Sentinel-2) and synthetic aperature radar (SAR) (Sentinel-1 and ALOS-PALSAR) sensors. The inclusion of the two SAR sensors allowed the use of regions of the electromagnetic spectrum that have been related to leaf density (Sentinel-1 C-band)^41–44^ and forest height (ALOS-PALSAR L-band)^45–47^, increasing the number of possible predictors and potentially reducing the error of the models. Each of these five national maps of forest structure was formed by a mosaic of regional maps corresponding to the five natural regions into which Colombia is divided. We did this to reduce errors in model predictions related to contrasting environmental conditions among regions, and relative uniformity within regions.

**Table 1:** Description of the five GEDI metrics selected for mapping.

Metric (GEDI name)	Abbreviation (units)	Definition
Canopy height (CH)	CH (m)	Relative height (RH) at the 95^th^ percentile of returned energy.
Height of half the accumulated energy (RH50)	RH50 (m)	Relative height (RH) at the 50^th^ percentile of returned energy.
Total cover (cover-a0)	COVER (%)	Total canopy cover, defined as the percentage of the ground covered by the vertical projection of canopy material
Foliage Height Diversity (fhd_normal)	FHD (Index)	Foliage height diversity index calculated by vertical foliage profile normalized by total plant area index, that is: *FHD* = − ∑*i Ni* × *log* (*Ni*) where *Ni* is the proportion of vertical LAI profile lies in the ith of the chosen horizontal layers.
Total Plant Area Index (pai)	PAI (Index-m^2^/m^2^)	One half of the total plant area projected per unit ground surface.

## Methods

### Study area

**Fig. 1:**
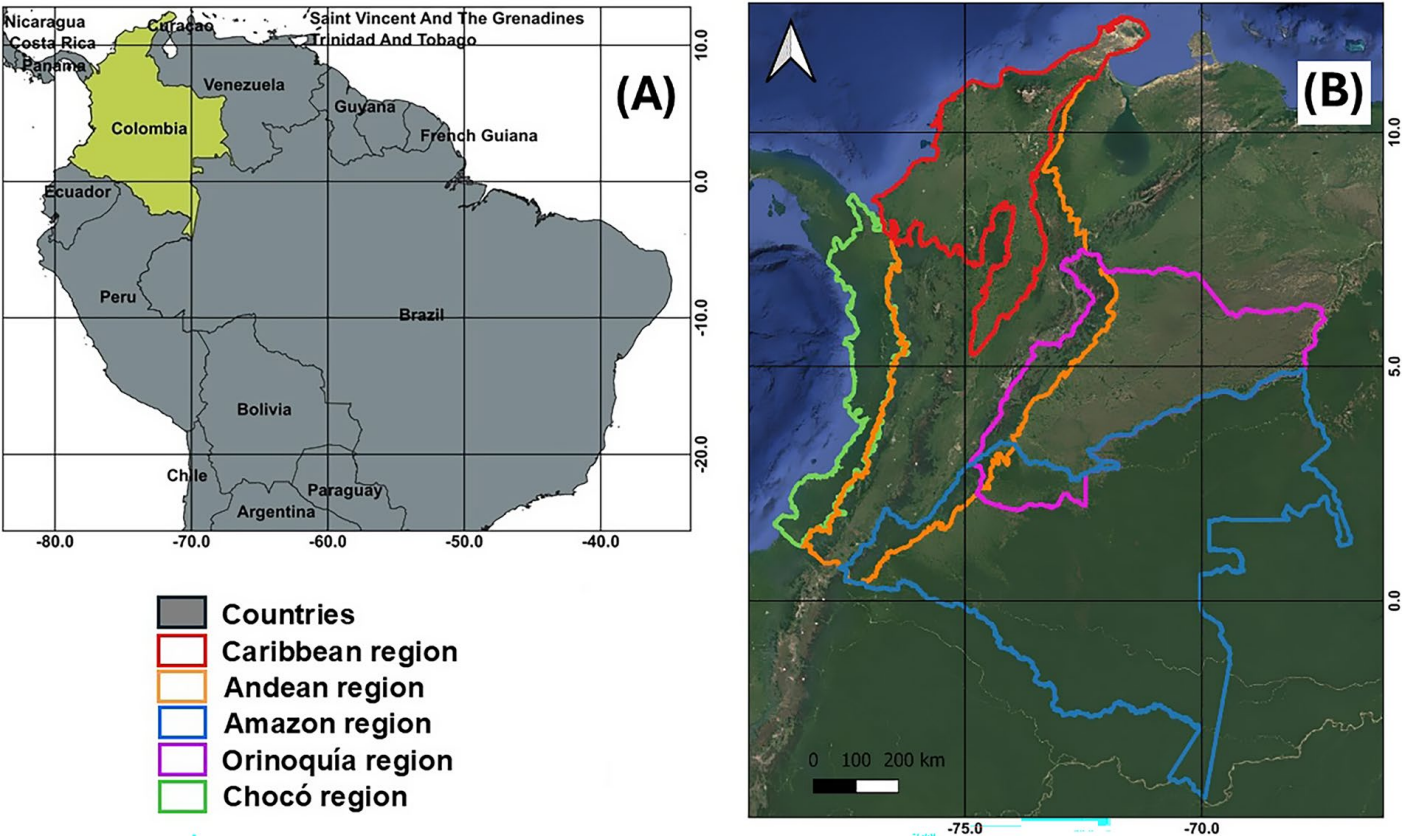
Colombian terrestrial land (**A**) and natural regions (**B**).

Colombia’s mainland territory presents an area of ~1.142 million km^2^ in the northwestern corner of South America. Colombia is categorized as a megadiverse country since it contains record high numbers in counts of several taxa (e.g., birds, mammals, amphibians, butterflies, freshwater fish, orchids, vascular plants), ecosystems, types of vegetation, and types of forests^48^. Colombian environmental authorities divide the country into five primary natural regions, Andean, Caribbean, Amazon, Chocó (Pacific), and Orinoquía (Fig. 1). It was estimated, in 2020, that 52.1% of Colombia is covered by forests distributed as follows: 64.8% in the Amazon, 17.2% in the Andes, 7.7% in Chocó, 5.5% in the Caribbean, and 4.8% in Orinoquía^49^. The Amazon is dominated by Tropical Moist-Forest, the Chocó by Tropical Rain-Forest, the Caribbean and Orinoquía by Tropical Dry Forest, and the Andes presents mosaics of Tropical Dry-Forest, Tropical Moist-Forest, and Tropical Rain-Forest, separated by small distances in some areas due to the high environmental variability generated by the branching of the Andes Mountain range into three mountain ranges (Western, Central, and Eastern Mountain ranges)^50^.

### GEDI response variables

We downloaded all the L2A and L2B granule data of GEDI (version 2.1) for the Colombian territory corresponding to the years 2019, 2020, and 2021 to build regional datasets of the five metrics (Table 1). High quality footprints were afterward selected using the comprehensive filtering process published by Burns *et al*.^3^. First, we selected quality shots that suitably estimated ground elevation and vegetation structure metrics. Selection criteria included minimal surface water, minimal urban cover, leaf on vegetation status, vegetation structure metrics within expected ranges, and ground elevation agreement with a reference DEM, among others. Then, we linked the filtered L2A, L2B, and L4A datasets by shot number. Finally, we used a dictionary of local outlier granules produced by University of Maryland to exclude orbit segments that were identified as local outliers, typically associated with low clouds. This quality-filtering procedure resulted in 5,720,940 high-quality footprints for the Amazon region, 5,620,920 for the Andean region, 5,584,260 for the Caribbean region, 5,630,300 for Orinoquía, and 1,105,860 for Chocó.

### SAR and multispectral predictors

We constructed 76 mosaics of temporal and textural metrics using the pixel values of all imagery of Sentinel-1 (SAR data of the c-band) and Sentinel-2 (multispectral data) available between 1 January 2019 and 31 December 2021 in Google Earth Engine – GEE^51^. By using all imagery of these three years in all our calculations, one year before and one year after 2020, we maximized the use of data for robust estimation, i.e. reduced error and uncertainty. The temporal metrics were average (X) and standard deviation (SD)^21,41,42^ while the textural metrics were sum average (SAVG) and difference variance (DVAR)^52^. These four metrics represented the central tendency (X and SAVG) and evaluated the data dispersion (SD and DVAR), generating balance among predictors. Textural metrics were calculated in neighborhoods of 3 × 3 pixels using the glcmTexture and map functions of GEE, which allowed us to estimate texture in each image of the temporal collection and later obtain an average. A description of the temporal and textural metrics is found in Table 2.

**Table 2:** Temporal and textural metrics.

Type of metrics	Name (Acronym)	Definition
Temporal	Temporal mean (X).	*X* $={\sum }_{1}^{n}x1$ */ n* Where: x = pixel value in the temporal sequence of images. n = number of pixels in the sequence of images
	Temporal standard deviation (SD).	*SD = √[Σ(x1 - X)² / n]* Where: X = Temporal mean x = pixel value in the temporal sequence of images. n = number of pixels in the sequence of images
Textural	Sum average (SAVG).	${SAVG}={\sum }_{i=2}^{2{Ng}}i$ *p*_*x+y*_^*i*^ Where: *Ng = *number of gray levels. i = row indices of the GLCM matrix (equal to gray level of reference cell) j = column indices of the GLCM matrix (equal to gray level of neighboring cell) x and y = pictorial information represented in two variables x and y.
	Difference variance (DVAR).	*DVAR = variance of p*_*x-y*_ Where: x and y = pictorial information represented in two variables x and y.

To develop the Sentinel-1 mosaics, the Sentinel-1 SAR GRD (C-band Synthetic Aperture Radar Ground Range Detected) product data sets^53^ were processed by applying an angular-based radiometric slope correction using a backscatter coefficient gamma nought, in addition to the calibration and ortho-correction of these data sets^54^. To develop the Sentinel-2 mosaics, we initially created image mosaics from Sentinel 2A surface reflectance products; however, because images were processed in tiles and bidirectional reflectance distribution function (BRDF) adjustments had not been applied, noticeable artifacts due to surface anisotropy and tile boundaries were present. To overcome this limitation, we applied a normalization approach based on the method outlined in Potapov *et al*. (2012) to Sentinel-2 Level 1 C top-of-atmosphere (TOA) imagery and constructed mosaics from these normalized images^55^. The approach reduces artifacts caused by surface anisotropy and variations in the viewing and solar geometries that remain in Sentinel-2A Level-2A products, resulting in mosaics with more consistent reflectance across scenes and acquisition dates. However, because the procedure adjusts TOA reflectance rather than performing full atmospheric correction, it does not provide true surface reflectance as other physics-based methods do. This method uses MODIS BRDF-adjusted reflectance as the normalization target. Here, we used a 10-year median of MODIS land surface reflectance bands, filtered to include only good-quality observations as indicated by the QA bands. We first selected relatively clear pixels from each image by using the scene classification map from the corresponding Sentinel 2 A SR product, which is developed by ESA and effectively removes most clouds and cloud shadows from L1C (Top-of-Atmosphere) and L2A (Surface Reflectance) imagery^56^. Next, the mean bias between MODIS and Sentinel-2 reflectance was calculated and used to adjust Sentinel-2 TOA reflectance, excluding pixels with large reflectance differences. To account for surface anisotropy, a linear regression between reflectance bias and distance from the center of each Sentinel-2 scene was applied to each spectral band independently. Table 3 shows the corresponding bands between the Sentinel-2 MSI and MODIS sensors used in the normalization process; however, there are no direct MODIS equivalents for the Sentinel-2 red edge bands. We generated synthetic MODIS red edge bands by modeling Sentinel-2 red edge bands as linear combinations of the MODIS red and near-infrared (NIR) bands. To do this, we convolved known surface reflectance spectra from the ECOSTRESS spectral library^57,58^ with the spectral response functions (SRFs) of the Sentinel-2 red edge bands and the MODIS red and NIR bands (SRFs obtained from the Pyspectral Python library^59^). The simulated reflectance values from the Sentinel-2 red edge bands served as dependent variables, while MODIS red and NIR band reflectances were used as independent variables.

**Table 3:** Sentinel 2 MSI (The MultiSpectral Imager) bands with analogous bands from the MODIS platform.

Sentinel-2 band	Central wavelength (nm)	MODIS band	Central wavelength (nm)
Blue	490	3	469
Green	560	4	555
Red	665	1	645
Red edge 1	705	—	—
Red Edge 2	740	—	—
Red Edge 3	783	—	—
NIR	842	2	859
NIR narrow	865	2	859
SWIR	1610	6	1640
SWIR	2190	7	2130

We also constructed six mosaics for ALOS-PALSAR data applying a variation to the previous methodology described for Sentinel. We first obtained two metrics for the two polarizations of ALOS-2-PALSAR data, the average of years 2019, 2020, and 2021 using the GEE product 25 m PALSAR/PALSAR-2 mosaic^47^, since this is a one-date annual product created by mosaicking imagery from PALSAR/PALSAR-2. We then obtained four textural metrics over the previous annual mean, SAVG and DVAR for each polarization, estimated in neighborhoods of 3 × 3 pixels. A summary of each backscatter coefficient, band, and index used to build the 82 mosaics for the Sentinel-1, Sentinel-2, and ALOS-2-PALSAR data is shown in Table 4 and scripts used to build these mosaics are available in the section Code availability.

**Table 4:** Summary of the multispectral and SAR data used to build temporal (average-X and standard deviations-SD) and textural (sum average-SAVG and difference variance-DVAR) metrics.

Satellite (data type)	Band, index name, or backscatter coefficient	Wavelength or definition
Sentinel-1 (SAR)	VV of C band	5.6 cm (5.405 GHz)
	VH of C band	5.6 cm (5.405 GHz)
	VH / VV of C band	5.6 cm (5.405 GHz)
	VV - VH of C band	5.6 cm (5.405 GHz)
Sentinel-2 (Multispectral)	Blue	492.1–496.6 nm
	Green	559–560 nm
	Red	664.5–665 nm
	Red edge 1	703.8-703.9 nm
	Red edge 2	739.1-740.2 nm
	Red edge 3	779.7-782.5 nm
	Near Infrared – NIR	835.1-833 nm
	Red edge 4	864-864.8 nm
	Short wave infrared 1 – SWIR1	1610.4-1613.7 nm
	Short wave infrared 2 – SWIR2	2185.7-2202.4 nm
	NDVI – Normalized Difference Vegetation Index	(NIR-Red)/(NIR + Red)
	EVI – Enhanced Vegetation Index	G * (NIR – Red)/(NIR + C1*RED-C2*Blue + L)
	SAVI – Soil Adjusted Vegetation Index	(1 + L)*(NIR-Red)/(NIR + Red + L)
	SVVI – Spectral variability vegetation index	SD(Blue, Green, Red, NIR, SWIR1, SWIR2) – SD(NIR, SWIR1, SWIR2)
	RNDVI- Red-edge Normalized Difference Vegetation Index	(NIR-RED_EDGE_2)/ (NIR + RED_EDGE_2)
ALOS-2-PALSAR (SAR)	HH of the L band	23.62 cm (1270 MHz)
	HV of the L band	23.62 cm (1270 MHz)

The first letter and second letter in the SAR data (H or V) refer to the transmit and return signals; H stands for horizontal and V for vertical polarizations.

#### Prediction and mapping

To construct maps of the five GEDI metrics that describe the structure of Colombian forests at the year 2020 (Table 1), we first built maps for each natural region for each GEDI metric and then mosaiced these regional maps to create final national maps. We used this mapping approach because each natural region tends to have some similarity in forest types and environmental conditions (e.g., climate, topography, altitude) which allowed us to control sources of error in spatial modeling^60,61^. Other approaches typically applied in remote sensing modeling of large areas, such as mapping throughout the entire study area^62^ or mapping across regular grids that cover the study area^35^, could combine different forest types and environmental conditions, increasing modeling errors, given the highly heterogeneous characteristics of the Colombian territory.

Each regional map was constructed using the numerical values of each GEDI metric as the response variable, the associated values of the temporal and textural SAR and multispectral metrics as predictors, and the Random Forest algorithm (RF)^63,64^. Although in most regions we identified more than 5 million high-quality GEDI footprints we randomly subsampled 1,200,000 of these footprints for each regional model. This is the approximate maximum number of observations that our high-performance computing system could process for RF modeling with 82 predictors. The Choco region did not require any sub-sampling as we identified 1,105,860 high-quality footprints there. We then tuned RF hyperparameters, including the number of variables randomly sampled as candidates at each split and minimum size of terminal nodes. Once the best regional model was identified, the regional map for each GEDI metric was built based on the 82 mosaics of the SAR and multispectral predictors mentioned previously. We used the R packages “randomForest”^64^ and “Caret”^65^ for the RF modeling, “Boruta”^66^ to apply the Boruta algorithm for feature selection, and “raster” for mapping^67^.

## Data Records

**Fig. 2:**
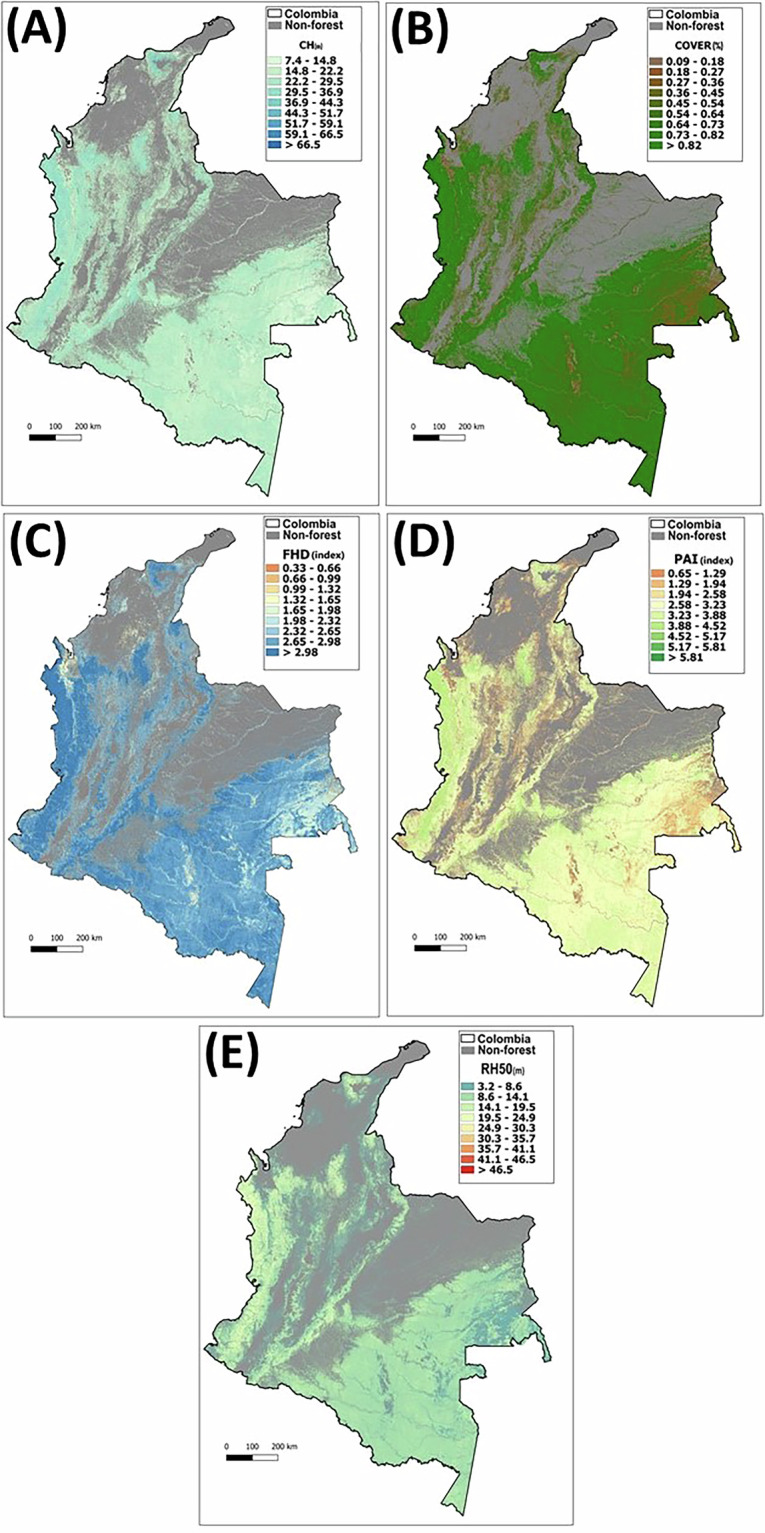
Maps of forest structure of Colombia in the year 2020. (**A**) Canopy height (CH) in meters, (**B**) Total cover (COVER) in percentage, (**C**) Foliage Height Diversity (FHD) in index, (**D**) Total Plant Area Index (PAI) in index-m^2^/m^2^, and (**E**) Height of half the accumulated energy (RH50) in meters.

Maps of Colombian forest vertical structure for the year 2020 (Fig. 2) are available to download in GeoTiff format in Zenodo^68^: https://zenodo.org/records/15493516. These maps are also accessible in Google Earth Engine in the links below, which are organized corresponding to a tile shapefile, where each map is split into eleven tiles, with tile numbering starting at one and running from left to right, top to bottom, starting at the top left. The shapefile consists of four rows and three columns but note that the top row has only two tiles as the upper right tile does not contain any forest pixels in Colombia.

Tile shapefile

https://code.earthengine.google.com/?asset=projects/ee-jantzenator/assets/colombia_forest_structure/COLOMBIA_FOREST_TILES

CH (canopy height)

https://code.earthengine.google.com/?asset=projects/ee-jantzenator/assets/colombia_forest_structure/ch/CH_COLOMBIA_FOREST_1

https://code.earthengine.google.com/?asset=projects/ee-jantzenator/assets/colombia_forest_structure/ch/CH_COLOMBIA_FOREST_2

https://code.earthengine.google.com/?asset=projects/ee-jantzenator/assets/colombia_forest_structure/ch/CH_COLOMBIA_FOREST_3

https://code.earthengine.google.com/?asset=projects/ee-jantzenator/assets/colombia_forest_structure/ch/CH_COLOMBIA_FOREST_4

https://code.earthengine.google.com/?asset=projects/ee-jantzenator/assets/colombia_forest_structure/ch/CH_COLOMBIA_FOREST_5

https://code.earthengine.google.com/?asset=projects/ee-jantzenator/assets/colombia_forest_structure/ch/CH_COLOMBIA_FOREST_6

https://code.earthengine.google.com/?asset=projects/ee-jantzenator/assets/colombia_forest_structure/ch/CH_COLOMBIA_FOREST_7

https://code.earthengine.google.com/?asset=projects/ee-jantzenator/assets/colombia_forest_structure/ch/CH_COLOMBIA_FOREST_8

https://code.earthengine.google.com/?asset=projects/ee-jantzenator/assets/colombia_forest_structure/ch/CH_COLOMBIA_FOREST_9

https://code.earthengine.google.com/?asset=projects/ee-jantzenator/assets/colombia_forest_structure/ch/CH_COLOMBIA_FOREST_10

https://code.earthengine.google.com/?asset=projects/ee-jantzenator/assets/colombia_forest_structure/ch/CH_COLOMBIA_FOREST_11

COVER (canopy cover)

https://code.earthengine.google.com/?asset=projects/ee-jantzenator/assets/colombia_forest_structure/cover/COVER_COLOMBIA_FOREST_1

https://code.earthengine.google.com/?asset=projects/ee-jantzenator/assets/colombia_forest_structure/cover/COVER_COLOMBIA_FOREST_2

https://code.earthengine.google.com/?asset=projects/ee-jantzenator/assets/colombia_forest_structure/cover/COVER_COLOMBIA_FOREST_3

https://code.earthengine.google.com/?asset=projects/ee-jantzenator/assets/colombia_forest_structure/cover/COVER_COLOMBIA_FOREST_4

https://code.earthengine.google.com/?asset=projects/ee-jantzenator/assets/colombia_forest_structure/cover/COVER_COLOMBIA_FOREST_5

https://code.earthengine.google.com/?asset=projects/ee-jantzenator/assets/colombia_forest_structure/cover/COVER_COLOMBIA_FOREST_6

https://code.earthengine.google.com/?asset=projects/ee-jantzenator/assets/colombia_forest_structure/cover/COVER_COLOMBIA_FOREST_7

https://code.earthengine.google.com/?asset=projects/ee-jantzenator/assets/colombia_forest_structure/cover/COVER_COLOMBIA_FOREST_8

https://code.earthengine.google.com/?asset=projects/ee-jantzenator/assets/colombia_forest_structure/cover/COVER_COLOMBIA_FOREST_9

https://code.earthengine.google.com/?asset=projects/ee-jantzenator/assets/colombia_forest_structure/cover/COVER_COLOMBIA_FOREST_10

https://code.earthengine.google.com/?asset=projects/ee-jantzenator/assets/colombia_forest_structure/cover/COVER_COLOMBIA_FOREST_11

FHD_PAI (foliage height diversity calculated from plant area index)

https://code.earthengine.google.com/?asset=projects/ee-jantzenator/assets/colombia_forest_structure/fhd_pai/FHD_PAI_COLOMBIA_FOREST_1

https://code.earthengine.google.com/?asset=projects/ee-jantzenator/assets/colombia_forest_structure/fhd_pai/FHD_PAI_COLOMBIA_FOREST_2

https://code.earthengine.google.com/?asset=projects/ee-jantzenator/assets/colombia_forest_structure/fhd_pai/FHD_PAI_COLOMBIA_FOREST_3

https://code.earthengine.google.com/?asset=projects/ee-jantzenator/assets/colombia_forest_structure/fhd_pai/FHD_PAI_COLOMBIA_FOREST_4

https://code.earthengine.google.com/?asset=projects/ee-jantzenator/assets/colombia_forest_structure/fhd_pai/FHD_PAI_COLOMBIA_FOREST_5

https://code.earthengine.google.com/?asset=projects/ee-jantzenator/assets/colombia_forest_structure/fhd_pai/FHD_PAI_COLOMBIA_FOREST_6

https://code.earthengine.google.com/?asset=projects/ee-jantzenator/assets/colombia_forest_structure/fhd_pai/FHD_PAI_COLOMBIA_FOREST_7

https://code.earthengine.google.com/?asset=projects/ee-jantzenator/assets/colombia_forest_structure/fhd_pai/FHD_PAI_COLOMBIA_FOREST_8

https://code.earthengine.google.com/?asset=projects/ee-jantzenator/assets/colombia_forest_structure/fhd_pai/FHD_PAI_COLOMBIA_FOREST_9

https://code.earthengine.google.com/?asset=projects/ee-jantzenator/assets/colombia_forest_structure/fhd_pai/FHD_PAI_COLOMBIA_FOREST_10

https://code.earthengine.google.com/?asset=projects/ee-jantzenator/assets/colombia_forest_structure/fhd_pai/FHD_PAI_COLOMBIA_FOREST_11

PAI (plant area index)

https://code.earthengine.google.com/?asset=projects/ee-jantzenator/assets/colombia_forest_structure/pai/PAI_COLOMBIA_FOREST_1

https://code.earthengine.google.com/?asset=projects/ee-jantzenator/assets/colombia_forest_structure/pai/PAI_COLOMBIA_FOREST_2

https://code.earthengine.google.com/?asset=projects/ee-jantzenator/assets/colombia_forest_structure/pai/PAI_COLOMBIA_FOREST_3

https://code.earthengine.google.com/?asset=projects/ee-jantzenator/assets/colombia_forest_structure/pai/PAI_COLOMBIA_FOREST_4

https://code.earthengine.google.com/?asset=projects/ee-jantzenator/assets/colombia_forest_structure/pai/PAI_COLOMBIA_FOREST_5

https://code.earthengine.google.com/?asset=projects/ee-jantzenator/assets/colombia_forest_structure/pai/PAI_COLOMBIA_FOREST_6

https://code.earthengine.google.com/?asset=projects/ee-jantzenator/assets/colombia_forest_structure/pai/PAI_COLOMBIA_FOREST_7

https://code.earthengine.google.com/?asset=projects/ee-jantzenator/assets/colombia_forest_structure/pai/PAI_COLOMBIA_FOREST_8

https://code.earthengine.google.com/?asset=projects/ee-jantzenator/assets/colombia_forest_structure/pai/PAI_COLOMBIA_FOREST_9

https://code.earthengine.google.com/?asset=projects/ee-jantzenator/assets/colombia_forest_structure/pai/PAI_COLOMBIA_FOREST_10

https://code.earthengine.google.com/?asset=projects/ee-jantzenator/assets/colombia_forest_structure/pai/PAI_COLOMBIA_FOREST_11

RH50 (height at which 50% of lidar energy is returned)

https://code.earthengine.google.com/?asset=projects/ee-jantzenator/assets/colombia_forest_structure/rh50/RH50_COLOMBIA_FOREST_1

https://code.earthengine.google.com/?asset=projects/ee-jantzenator/assets/colombia_forest_structure/rh50/RH50_COLOMBIA_FOREST_2

https://code.earthengine.google.com/?asset=projects/ee-jantzenator/assets/colombia_forest_structure/rh50/RH50_COLOMBIA_FOREST_3

https://code.earthengine.google.com/?asset=projects/ee-jantzenator/assets/colombia_forest_structure/rh50/RH50_COLOMBIA_FOREST_4

https://code.earthengine.google.com/?asset=projects/ee-jantzenator/assets/colombia_forest_structure/rh50/RH50_COLOMBIA_FOREST_5

https://code.earthengine.google.com/?asset=projects/ee-jantzenator/assets/colombia_forest_structure/rh50/RH50_COLOMBIA_FOREST_6

https://code.earthengine.google.com/?asset=projects/ee-jantzenator/assets/colombia_forest_structure/rh50/RH50_COLOMBIA_FOREST_7

https://code.earthengine.google.com/?asset=projects/ee-jantzenator/assets/colombia_forest_structure/rh50/RH50_COLOMBIA_FOREST_8

https://code.earthengine.google.com/?asset=projects/ee-jantzenator/assets/colombia_forest_structure/rh50/RH50_COLOMBIA_FOREST_9

https://code.earthengine.google.com/?asset=projects/ee-jantzenator/assets/colombia_forest_structure/rh50/RH50_COLOMBIA_FOREST_10

https://code.earthengine.google.com/?asset=projects/ee-jantzenator/assets/colombia_forest_structure/rh50/RH50_COLOMBIA_FOREST_11

## Data Overview

The five national Maps of forest vertical structure for Colombia presented in this publication correspond to the 2020 year (Fig. 2), have the coordinate reference system EPSG:4326, spatial resolution of 25 m, and the data type Float32. Forest areas were identify by masking out all areas with <70% tree cover based on Hansen Global Forest Change database v1.12 (2000–2024)^34^. The map of Canopy height (CH) is in meters, the map of total cover (COVER) in percentage of cover, the map of Foliage Height Diversity (FHD) in the FHD index, the map of Total Plant Area Index (PAI) in the PAI index, and the map of height of half the accumulated energy (RH50) is in meters. The details of how map units were calculated are described in Table 1.

## Technical Validation

**Fig. 3:**
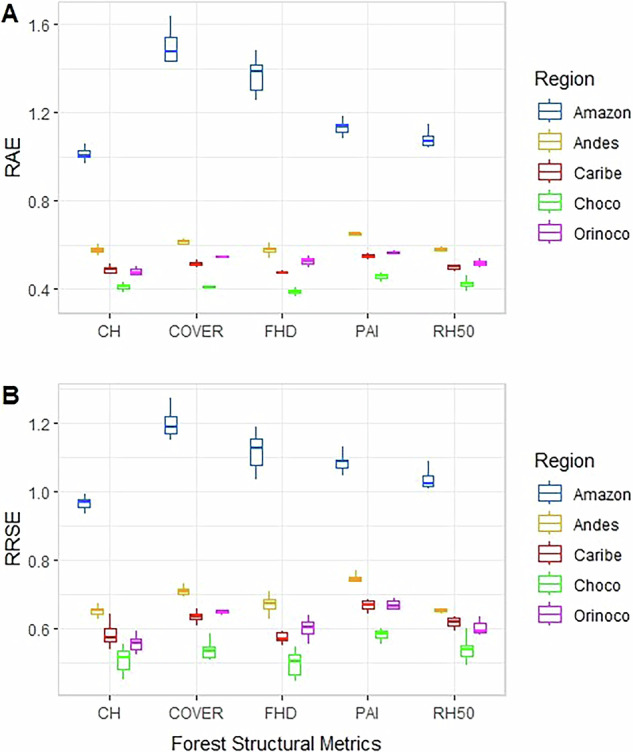
Error estimation, RAE (Relative Absolute Error) and RRSE (Root Relative Squared Error), of forest the forest structural maps of Colombia.

We implemented three types of validation: 1) cross validation using sample data (VSD), 2) validation using external data (VED), and 3) Validation testing the interrelationship curves of the forest structural variables between footprint data vs. predicted data (VRC). VSD refers to error estimates calculating two metrics that allow comparisons between different units, RAE (Relative Absolute Error) and RRSE (Root Relative Squared Error), and two error metrics for absolute data to recognize the magnitude of the error, MAE (Mean Absolute Error) and RMSE (Root Mean Squared Error). These four-error metrics were calculated by sampling data partitions, using the sample data (GEDI footprints) where 70% of the footprints were used for building the maps and 30% were used for testing the resulting maps. These validations using sample data were estimated in each regional map for each of the five-forest structural metrics applying resampling of 5000 on the testing data to estimate value-ranges. We found error differences among the natural regions; the Amazon and Andean regions tended to present the highest RAE and RRSE values (Fig. 3) with maximum RMSE magnitudes of ~5.8 m for CH, ~0.25 for COVER, ~0.42 for FHD, 1.69 of m^2^/m^2^ for PAI, and ~5.4 m for RH50 (Table 5).

**Table 5:** Error-estimates of the Validation using Sample Data (VSD) for the regional maps of forest structural metrics using MAE (Mean Absolute Error) and RMSE (Root Mean Squared Error).

Region	Forest structural metrics (units)	Range values		MAE		RMSE	
		min	max	mean	SD	mean	SD
Amazon Reg.	CH (m)	1.79	79.25	4.60	0.16	5.86	0.36
	COVER (%)	0.01	0.97	0.21	0.00	0.25	0.00
	FHD (index)	0.63	3.36	0.34	0.01	0.42	0.02
	PAI (index)	0.01	7.38	1.39	0.03	1.69	0.03
	RH50 (m)	0	36.98	4.38	0.12	5.04	0.11
Andean Reg.	CH (m)	0.2	83.1	3.96	0.16	5.68	0.25
	COVER (%)	0	0.97	0.17	0.00	0.23	0.00
	FHD (index)	0	3.36	0.24	0.01	0.37	0.02
	PAI (index)	0	7.35	1.08	0.03	1.40	0.03
	RH50 (m)	0	38.57	2.92	0.13	3.71	0.19
Caribbean Reg.	CH (m)	1.6	73.5	3.27	0.14	4.83	0.49
	COVER (%)	0	0.97	0.16	0.00	0.22	0.01
	FHD (index)	0	3.35	0.24	0.00	0.36	0.01
	PAI (index)	0	7.19	0.92	0.03	1.26	0.04
	RH50 (m)	0	33.38	2.22	0.09	3.17	0.15
Chocó Reg.	CH (m)	0.03	89.04	2.32	0.12	4.08	0.40
	COVER (%)	0.01	0.97	0.09	0.00	0.14	0.01
	FHD (index)	0	3.37	0.12	0.00	0.23	0.02
	PAI (index)	0	7.38	0.66	0.02	1.01	0.03
	RH50 (m)	0	32.56	1.81	0.17	2.95	0.29
Orinoquía Reg.	CH (m)	1.76	62.61	2.17	0.11	3.18	0.23
	COVER (%)	0.01	0.97	0.15	0.00	0.21	0.00
	FHD (index)	0	3.37	0.22	0.01	0.34	0.02
	PAI (index)	0	7.38	0.93	0.01	1.25	0.02
	RH50 (m)	0	32.56	1.92	0.07	2.56	0.11
Global Colombia.	CH (m)	1.72	89	5.33	0.29	7.41	0.71
	COVER (%)	0	0.98	0.24	0.00	0.31	0.00
	FHD (index)	0.66	3.38	0.37	0.01	0.46	0.01
	PAI (index)	0	8.05	1.53	0.01	1.82	0.01
	RH50 (m)	0	38.54	4.56	0.11	5.62	0.18

We added the erros estimated for the entire Colombia to observe the error reduction of our regional approach.

VED refers to error estimates using GEDI footprints simulated using 578 km^2^ of discrete ALS-LiDAR across the Chocó natural region. A description of this data set can be found at Fagua *et al*.^21^. We followed the approach described by Hancock *et al*.^69^ to simulate GEDI footprints using the ALS-LiDAR data and to estimate values of CH, RH50, FHD, and COVER. We note that PAI could not be simulated due to the lack of some parameters necessary for its estimation. The process to simulate the GEDI footprints first consisted of noise removal using the Statistical Outlier Removal method of the R package lasR^70^. Next, we established a grid with the same resolution as the vertical structure maps. We later identified the centroid of each raster cell that was contained within one of the LiDAR tiles to derive a simulated GEDI footprint and its corresponding CH, RH50, FHD, and COVER values, using the Rgedisimulator tool of the R package rGEDI^71^. We finally estimated the same error metrics described above, RAE, RRSE, MAE and RMSE, by comparing 5000 resamples of CH, RH50, FHD, and COVER simulated-values with the corresponding values from the resulting maps in the Choco. Simulated GEDI footprints were selected randomly using a spatial filter of 200 m. Parameters and scripts of GEDI simulation using ALS-LiDAR can be found at the github site for this manuscript (see Code Availability). We found higher errors for the VED validation compared with VSD validation in the Choco (Table 6). This was expected since error estimates from ALS-LiDAR can be considered field validation^24,35,72,73^, which usually results in higher errors compared with errors estimated by cross-validation with reserved sample data.

**Table 6:** Error-estimates of the Validation using External Data (VED) for the Choco maps calculating RAE (Relative Absolute Error), RRSE (Root Relative Squared Error), MAE (Mean Absolute Error) and RMSE (Root Mean Squared Error).

Forest structural metrics	RAE		RRSE		MAE		RMSE	
	mean	SD	mean	SD	mean	SD	mean	SD
CH (m)	0.5	0.03	0.64	0.03	6.49	0.14	8.65	0.25
COVER (%)	0.61	0.06	0.67	0.05	0.24	0.01	0.32	0.01
FHD (index)	0.51	0.015	0.62	0.08	1.31	0.02	1.4	0.03
PAI (index)	—	—	—	—	—	—	—	—
RH50 (m)	0.55	0.01	0.67	0.01	5.78	0.08	6.83	0.11

**Fig. 4:**
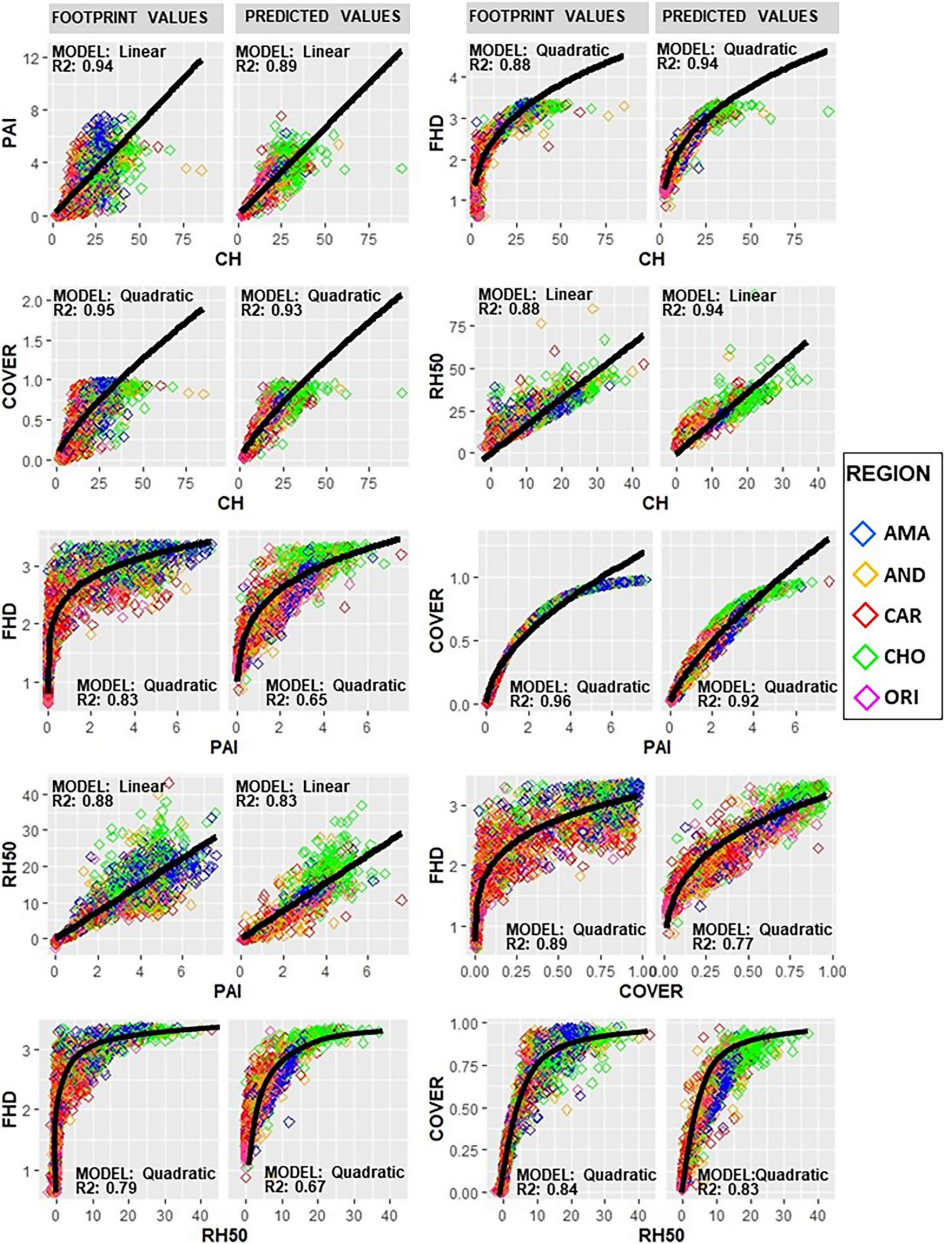
Interrelationship curves of the forest structural variables for footprint and predicted data.

Finally, VRC evaluates the extent to which interrelationship curves between the sample data (our five metrics of GEDI footprints that describe forest structure) are preserved in the predicted data (mapped pixel data)^21^. Forest structure metrics covary (see Footprint values of Fig. 4); it is therefore important evaluate whether our independent models preserve the interrelationships of forest metrics as observed by GEDI. We consider this approach a useful complement to typical procedures because it indicates to users that even though the metrics were modeled independently, the predicted values reproduce observed relationships between structure metrics that may be important for forest ecology and conservation. We randomly selected 5000 footprints and 5000 pixels in the resulting maps to compare the interrelationship curves among the metrics. These 5000 pixels of predicted data did not coincide with the locations of the footprints. We observe that interrelationship curves and their parameters between the variable pairs of the footprints were maintained in the mapped pixel data (Fig. 4 and Table 7).

**Table 7:** Regression models for the interrelationship between pairs of forest structural variables using GEDI footprints and predicted pixel values.

Regression model		GEDI Footprints values			Predicted pixel values		
Type	Model	R^2^	P value	F value	R^2^	P value	F value
Linear	PAI~CH	0.94	2.2e-16	5416	0.89	2.2e-16	14700
Quadratic	FHD~CH	0.88	2.2e-16	8879	0.94	2.2e-16	27580
Quadratic	COVER~CH	0.95	2.2e-16	27580	0.93	2.2e-16	18300
Linear	RH50~CH	0.88	2.2e-16	11610	0.85	2.2e-16	25790
Quadratic	FHD~PAI	0.83	2.2e-16	4320	0.65	2.2e-16	12720
Quadratic	COVER~PAI	0.96	2.2e-16	31530	0.92	2.2e-16	68340
Quadratic	RH50~PAI	0.88	2.2e-16	12900	0.83	2.2e-16	24500
Quadratic	FHD~COVER	0.89	2.2e-16	7528	0.77	2.2e-16	23720
Quadratic	FHD~RH50	0.79	2.2e-16	4686	0.67	2.2e-16	10600
Quadratic	COVER~RH50	0.84	2.2e-16	10820	0.83	2.2e-16	18280

## Usage Notes

Since the five produced maps correspond to Essential Biodiversity Variables, at moderately high spatial resolution (25 m) and, provide coverage throughout the continental territory of Colombia, they can be used to monitor and map the state of biodiversity and other environmental variables across the country. Previous works show that similar forest structural metrics have allowed precise mapping of tree alpha diversity, carbon content, and forest degradation, among others^21,39,74,75^. We note the regional approach in the creation of the maps accounts for the natural environmental variation of Colombia’s forests, in addition to reducing errors, which thereby provides more representative maps compared to global estimates that calculate without regional distinctions or are developed at lower spatial resolutions. Another point to highlight is that our maps were made for the forest areas of Colombia for the year 2020, using sample data for forested areas only. Forest areas were identify based on Hansen Global Forest Change database v1.12 (2000–2024)^34^. By focusing on forest cover type, we sought to reduce uncertainty for forest specific applications, such as mapping of forest diversity, carbon stock estimation, or forest degradation. These five national maps of forest structural metrics were formed by mosaicking of the regional maps using the average of the values in the transition zones. Although this method is commonly used in this type of analysis, possible unrepresentative values might be found in transition zones.

We note the error estimates of our CH maps in the Amazon and Andean regions, where errors were highest, are similar to the error estimates of an existing global CH map^35^ while the error estimates in other regions, such as Caribbean and Orinoquía, were lower than reported in such maps (Table 5). This, combined with the reported validations, indicates our maps are appropriate for forest assessments and related applications in Colombia.

## Data Availability

Resulting maps of this research are publicly accessible on Zenodo: https://zenodo.org/records/15493516.

## Code availability

The code is publicly accessible on Github^76^: https://github.com/CamiloFaguaUNAL/Forest_Structure_Colombia.
